# S-1 maintenance chemotherapy following definitive chemoradiotherapy in high-risk locally advanced nasopharyngeal carcinoma: a propensity score-matched analysis

**DOI:** 10.3389/fimmu.2025.1703844

**Published:** 2025-11-26

**Authors:** Quan Zuo, Jieqi Jia, Rong Liu, Jingsheng Zhao, Hui Wu, Jia Chen, Hexin Duan, Zaichuan Yang, Jing Shi, Qingling Yang, Fanglue Li, Zhibi Xiang, Chunhai Huang, Li Xiong, Zhi Yang

**Affiliations:** 1 Department of Oncology, People’s Hospital of Xiangxi Tujia and Miao Autonomous Prefecture, First Affiliated Hospital of Jishou University, Jishou, Hunan, China; 2 Department of Otolaryngology, People’s Hospital of Xiangxi Tujia and Miao Autonomous Prefecture, First Affiliated Hospital of Jishou University, Jishou, Hunan, China; 3 Department of Neurosurgery, People’s Hospital of Xiangxi Tujia and Miao Autonomous Prefecture, First Affiliated Hospital of Jishou University, Jishou, Hunan, China

**Keywords:** nasopharyngeal carcinoma, radical chemoradiotherapy, S-1, maintenance chemotherapy, a propensity score-matched analysis

## Abstract

**Objective:**

To evaluate the efficacy and safety of S-1 maintenance chemotherapy after definitive chemoradiotherapy in patients with high-risk, locally advanced nasopharyngeal carcinoma (NPC).

**Methods:**

A total of 536 patients with locally advanced NPC admitted to our hospital between August 2019 and November 2022 were screened. Of these, 171 met the inclusion criteria, including 93 patients who received S-1 maintenance therapy (experimental group) and 78 patients who did not receive maintenance therapy (control group). After 1:1 propensity score matching, 126 patients (63 per group) were finally included in the matched cohort for analysis. All patients received two to three cycles of TPF (paclitaxel liposome, cisplatin, and fluorouracil) neoadjuvant chemotherapy, followed by concurrent chemoradiotherapy within 1–2 weeks later.

**Results:**

Following treatment, survival outcomes were assessed. The 3-year progression-free survival rates were 87.3% in the experimental group versus 73.9% in the control group (*p* = 0.083). The 3-year locoregional recurrence-free survival (LRRFS) rates were 92.7% versus 82.4% (*p* = 0.277), respectively. The 3-year distant metastasis-free survival (DMFS) rate was significantly higher in the experimental group than in the control group (91.4% vs. 73.9%, *p* = 0.023). Similarly, the 3-year overall survival (OS) rate was 96.8% versus 82.1% (*p* = 0.035) in the experimental and control groups, respectively. Subgroup analysis revealed significant DMFS benefits with S-1 maintenance therapy in patients aged <60 years, male patients, those with stage IVa disease, and those with T3–T4 or N2–N3 classification. Additionally, patients who were Epstein-Barr virus (EBV) DNA-negative and those who achieved partial response or stable disease after definitive chemoradiotherapy also showed significant DMFS improvement. Patients with non-keratinizing carcinoma or EBV DNA positivity showed a trend toward improved DMFS as well.Safety:S-1 maintenance chemotherapy demonstrated a favorable safety profile, with only 3.2% of patients experiencing grade 3 adverse events.

**Conclusion:**

S-1 maintenance chemotherapy after definitive chemoradiotherapy significantly improves DMFS and OS in patients with high-risk locally advanced NPC, with a favorable safety profile.

## Introduction

Nasopharyngeal carcinoma (NPC) is a common head and neck malignancy with a distinct and uneven geographical distribution. Its highest incidence is reported in Southeast Asia, East Asia, and North Africa (1), with southern provinces of China, such as Guangdong, Guangxi, Fujian, and Hunan, showing particularly high prevalence (2). Over 70% of patients with NPC are diagnosed at a locally advanced stage (3). The introduction of intensity-modulated radiotherapy (IMRT), neoadjuvant chemotherapy, and concurrent chemoradiotherapy has significantly improved outcomes, achieving 5-year overall survival (OS) rates of 70%–85% for locally advanced NPC (4).

However, patients with high-risk features, such as N3 classification, T4N2M0 stage, positive post-treatment Epstein-Barr virus (EBV) DNA levels, or failure to achieve complete response after treatment, continue to face poor prognoses and a high risk of recurrence and metastasis. Patients initially diagnosed with N3 locally advanced NPC have a 5-year survival rate of only 50.4% (5), with distant metastasis being the primary cause of treatment failure (6–8). Therefore, improving outcomes for this high-risk population remains a critical challenge in NPC research.

Maintenance chemotherapy, which involves continued administration of effective single-agent chemotherapy after initial treatment response, aims to delay disease progression while preserving quality of life (9). Specifically, this approach was first established in the treatment of hematologic malignancies and has since been applied to advanced metastatic solid tumors, such as lung, pancreatic, and colorectal cancers (9–11). In metastatic NPC, maintenance chemotherapy has been associated with improved overall survival (12), prompting interest in its potential benefits for high-risk patients.

S-1 is an oral fluoropyrimidine derivative composed of tegafur, gimeracil, and oteracil potassium in a specific molar ratio (13). It demonstrates potent antitumor activity, is conveniently administered orally, has low toxicity, good tolerability, and provides survival benefits (14). Previous studies have confirmed that S-1 monotherapy as maintenance chemotherapy can improve survival outcomes in locally advanced NPC (12, 15).

This study aimed to evaluate the efficacy and safety of S-1 maintenance chemotherapy following definitive chemoradiotherapy in patients with high-risk locally advanced NPC. The findings provide evidence-based medical guidance for optimizing treatment strategies in this high-risk population.

## Materials and methods

### General data

**Figure 1:**
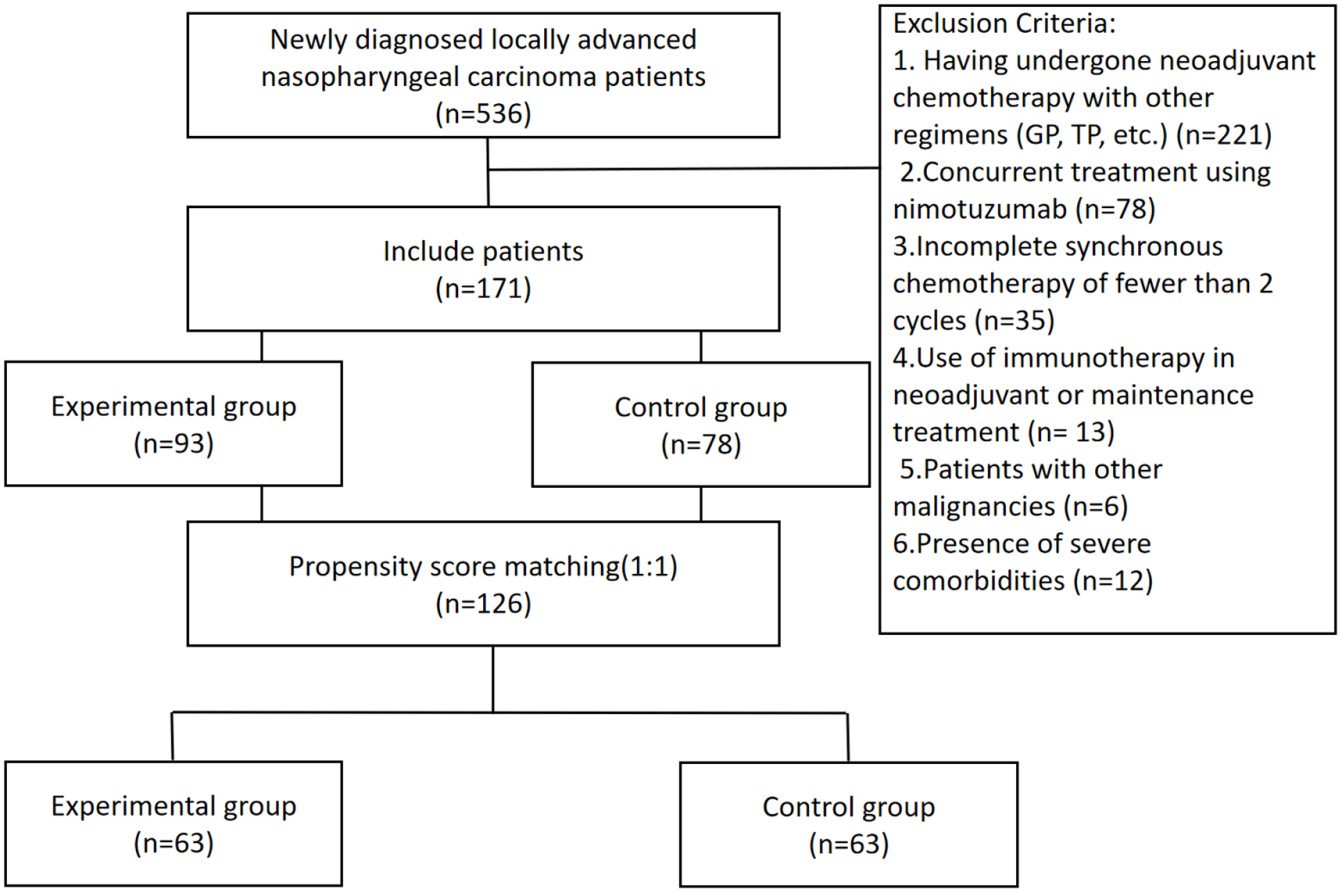
Flowchart of patient inclusion. GP, gemcitabine + cisplatin; TP, paclitaxel + cisplatin.

A total of 536 patients diagnosed with locally advanced NPC and admitted to our hospital between August 2019 and November 2022 were screened. Of these, 171 met the inclusion criteria, comprising 93 and 78 patients in the experimental and control groups, respectively. After 1:1 propensity score matching (PSM) based on covariates including age, sex, pathological types, clinical stage, T stage, N stage, EBV-DNA level after definitive chemoradiotherapy, and treatment response post-chemoradiotherapy, 126 patients (63 per group) were included in the final analysis (**Figure 1**). This study was approved by the ethics committee of our hospital. Patients and their families agreed to this study.

Inclusion criteria: (1) patients aged between 18 and 70 years; (2) pathologically confirmed NPC, including non-keratinizing carcinoma (differentiated or undifferentiated) and keratinizing squamous cell carcinoma (excluding neuroendocrine carcinoma); (3) stages III–IVA disease according to the AJCC 8th edition staging system based on imaging studies, excluding T3–4N0M0 and T3N1M0 stages.

Exclusion criteria: (1) having undergone neoadjuvant chemotherapy with other regimens (gemcitabine + cisplatin, paclitaxel + cisplatin, etc.); (2)concurrent treatment using nimotuzumab; (3) incomplete synchronous chemotherapy of fewer than 2 cycles; (4)use of immunotherapy in neoadjuvant or maintenance treatment; (5)patients with other malignancies, such as two primary cancers; (6) presence of severe comorbidities (such as severe cardiovascular or cerebrovascular diseases) that may affect patient survival.

### Treatment methods

All patients received neoadjuvant chemotherapy with liposomal paclitaxel, cisplatin, and fluorouracil (TPF) regimen, followed by definitive concurrent chemoradiotherapy. After completing chemoradiotherapy, patients were divided into the experimental group (with S-1 maintenance therapy) and the control group (without maintenance therapy).

#### Neoadjuvant chemotherapy

Patients received 2–3 cycles of TPF neoadjuvant chemotherapy before chemoradiotherapy (liposomal paclitaxel 135 mg/m² on d1, cisplatin 25 mg/m² on d1–3, and fluorouracil 600 mg/m² continuous intravenous infusion from d1–5), repeated every 3 weeks.

#### Concurrent chemoradiotherapy

Following neoadjuvant chemotherapy, patients proceeded to concurrent chemoradiotherapy, which included radiotherapy and chemotherapy components. Radiotherapy: Intensity-modulated radiotherapy (IMRT) was administered as follows: 95% PGTVnx (primary gross tumor volume of nasopharynx), 69.96–71.94 Gy/33 fractions; 95% PGTVnd (gross tumor volume of involved lymph nodes), 69.96 Gy/33 fractions; 95% PTV1 (high-risk planning target volume), 60.06 Gy/33 fractions; and 95% PTV2 (low-risk planning target volume), 50.4 Gy/28 fractions. Chemotherapy: Single-agent cisplatin (100 mg/m²), administered over 3 consecutive days every 21 days for 2 cycles during radiotherapy.

#### Maintenance chemotherapy

One month after completing chemoradiotherapy, patients were evaluated and divided into experimental and control groups based on whether they received S-1 maintenance therapy.

Experimental group: Patients received oral S-1 twice daily after meals for 14 consecutive days every 4 weeks. The S-1 dose was determined using the body surface area (BSA): BSA < 1.25 m^2^, 40 mg twice daily; 1.25 m^2^≤BSA<1.5 m^2^, 50 mg twice daily; and BSA≥1.5 m^2^, 60 mg twice daily. S1 maintenance chemotherapy was administered for up to 12 cycles, or treatment was terminated earlier in cases of disease progression, intolerable toxicity, or at the patient’s request. Dose adjustments were made according to the severity of adverse reactions. If a patient experienced an adverse event, the next cycle was delayed until the toxicity subsided to grade 1 or below. If grade ≥ 3 hematological or non-hematological toxicity occurred, or if grade ≥ 2 non-hematological toxicity recurred, the dose was reduced from 60 mg to 50 mg twice daily. If severe toxicity (grade ≥ 3) persisted despite this dose reduction, a further reduction to 40 mg twice daily was performed. If the patient could not tolerate 40 mg or experienced grade 4 toxicity, S-1 was discontinued.

Control group: Meanwhile, Patients received routine follow-up and observation only.

### Efficacy and observation endpoints

The primary endpoint was distant metastasis-free survival (DMFS), defined as the time from initiation of neoadjuvant chemotherapy to distant metastasis or death from any cause. The secondary endpoints were progression-free survival (PFS), locoregional recurrence-free survival (LRRFS), overall survival (OS), and toxicity. PFS was defined as the time from initiation of neoadjuvant chemotherapy to tumor progression or death from any cause. LRRFS was defined as the time from initiation of neoadjuvant chemotherapy to local recurrence or death from any cause. OS was defined as the time from initiation of neoadjuvant chemotherapy to death from any cause. Toxicity was graded using the Common Terminology Criteria for Adverse Events (CTCAE) version 5.0.

### Follow-up observation

Patients were followed up every 3 months during the first 2 years, every 6 months from years 2 to year 5, and annually thereafter. The final follow-up was on July 31, 2024.The follow-up duration ranged from 20 to 60 months, with a median of 37.6 months.

### Statistical analysis

SPSS version 26.0 was used for propensity score matching (PSM) with a caliper value of 0.02 and for statistical analysis. Survival differences were assessed using univariate analysis, the log-rank test, and multivariate analysis using the Cox proportional hazards model. Categorical data, including baseline characteristics and adverse events, were compared using the chi-square test, the Yates continuity correction, or Fisher’s exact test, as appropriate. Statistical significance was set at *P* < 0.05.

## Results

### Comparison of baseline characteristics between the two groups

Before propensity score matching (PSM), significant differences were found between the experimental and control groups in age and treatment efficacy after radical chemoradiotherapy (P < 0.05), while no differences were noted in sex, pathological type, clinical stage, T stage, N stage, or Epstein-Barr virus (EBV) DNA levels. Following PSM, these variables—including age, sex, pathological type, clinical stage, T stage, N stage, EBV DNA levels, and treatment efficacy—showed no significant differences between the groups (**Table 1**).

**Table 1:** Characteristics of the original and PSM cohorts.

Characteristics	Original cohort		*P*	Propensity-score matched cohort		*P*
	Experimental group (n=93) (%)	Control group (n=78) (%)		Experimental group (n=63) (%)	Control group (n=63) (%)	
Age(years)			<0.001			0.818
<60	79 (84.9)	62 (79.5)		51 (81.0)	52 (82.5)	
≥60	14 (15.1)	16 (20.5)		12 (19.0)	11 (17.5)	
Gender			0.511			0.148
Male	72 (77.4)	57 (73.1)		44 (69.8)	51 (81.0)	
Female	21 (22.6)	21 (26.9)		19 (30.2)	12 (19.0)	
Pathologic type			0.366			0.729
Non-keratinizing carcinoma	87 (93.5)	70 (89.7)		58 (92.1)	59 (93.7)	
Keratocarcinoma	6 (6.5)	8 (10.3)		5 (7.9)	4 (6.3)	
Clinical stage			0.325			0.364
III	18 (19.4)	20 (25.6)		10 (15.9)	14 (22.2)	
IVa	75 (80.6)	58 (74.4)		53 (84.1)	49 (77.8)	
T stage			0.309			0.998
T1	3 (3.2)	6 (7.7)		2 (3.2)	2 (3.2)	
T2	4 (4.3)	6 (7.7)		4 (6.3)	4 (6.3)	
T3	35 (37.6)	22 (28.2)		19 (30.2)	18 (28.6)	
T4	51 (54.8)	44 (56.4)		38 (60.3)	39 (61.9)	
N stage			0.581			0.762
N0	0 (0.0)	0 (0.0)		0 (0.0)	0 (0.0)	
N1	2 (2.2)	3 (3.8)		2 (3.2)	1 (1.6)	
N2	44 (47.3)	41 (52.6)		30 (47.6)	33 (52.4)	
N3	47 (50.5)	34 (43.6)		31 (49.2)	29 (46.0)	
EB-DNA (Copies/ml)			0.062			1.000
<100	90 (96.8)	70 (89.7)		61 (96.8)	60 (95.2)	
≥100	3 (3.2)	8 (10.3)		2 (3.2)	3 (4.8)	
Efficacy Evaluation			0.034			0.925
CR	49 (52.7)	56 (71.8)		45 (71.4)	43 (68.3)	
PR	43 (46.2)	21 (26.9)		17 (27.0)	19 (30.1)	
SD	1 (1.1)	1 (1.3)		1 (1.6)	1 (1.6)	

1. EB-DNA refers to the test results after radical chemoradiotherapy, with a level of <100 copies/ml considered negative and ≥100 copies/ml considered positive. 2. Efficacy evaluation refers to the assessment conducted after radical chemoradiotherapy.

### Comparison of efficacy between the two groups

Among patients receiving S-1 maintenance chemotherapy, 14 patients (22.2%) required DR: 10 (15.9%) had a one-level dose reduction and 4 (6.3%) had a two-level dose reduction. Four patients discontinued treatment (one due to nasopharyngeal necrosis, one due to distant metastasis, one due to grade III bone marrow suppression, and one due to patient request). Finally, 59 patients (93.7%) in the experimental group completed the 1-year treatment period.

**Figure 2:**
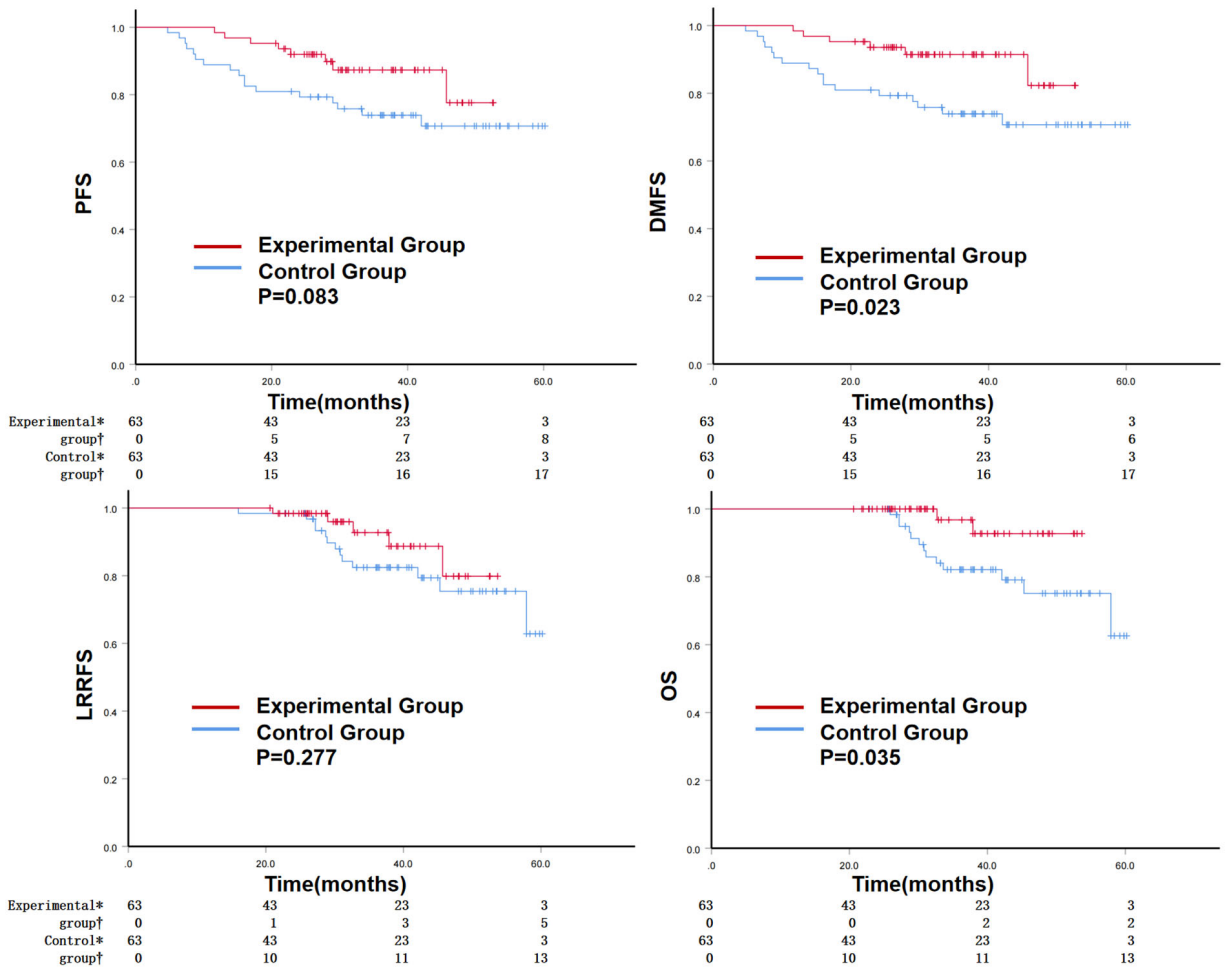
The Kaplan–Meier curves for PFS, DMFS, LRRFS, and OS after PSM. PFS, progression-free survival; DMFS, distant metastasis-free survival; LRRFS, locoregional recurrence-free survival; OS, overall survival; PSM, propensity score matching. *indicates the number of cases where events did not occur, † indicates the number of cases where events occurred.

The 3-year PFS rates of the experimental and control groups were 87.3% and 73.9%, (*P* = 0.083). The 3-year LRRFS rates of the experimental and control groups were 92.7% and 82.4%, (*P* = 0.277). The 3-year DMFS rate (91.4% vs. 73.9%, *P* = 0.023) and 3-year OS rate (96.8% vs. 82.1%, *P* = 0.035) were significantly higher in the experimental group than in the control group (**Figure 2**). Regarding disease progression, in the experimental group, there were eight cases of disease progression, two local recurrences, six distant metastases, and two deaths. In the control group, there were 17 cases of disease progression, two local recurrences, 17 distant metastases, and 13 deaths.

**Figure 3:**
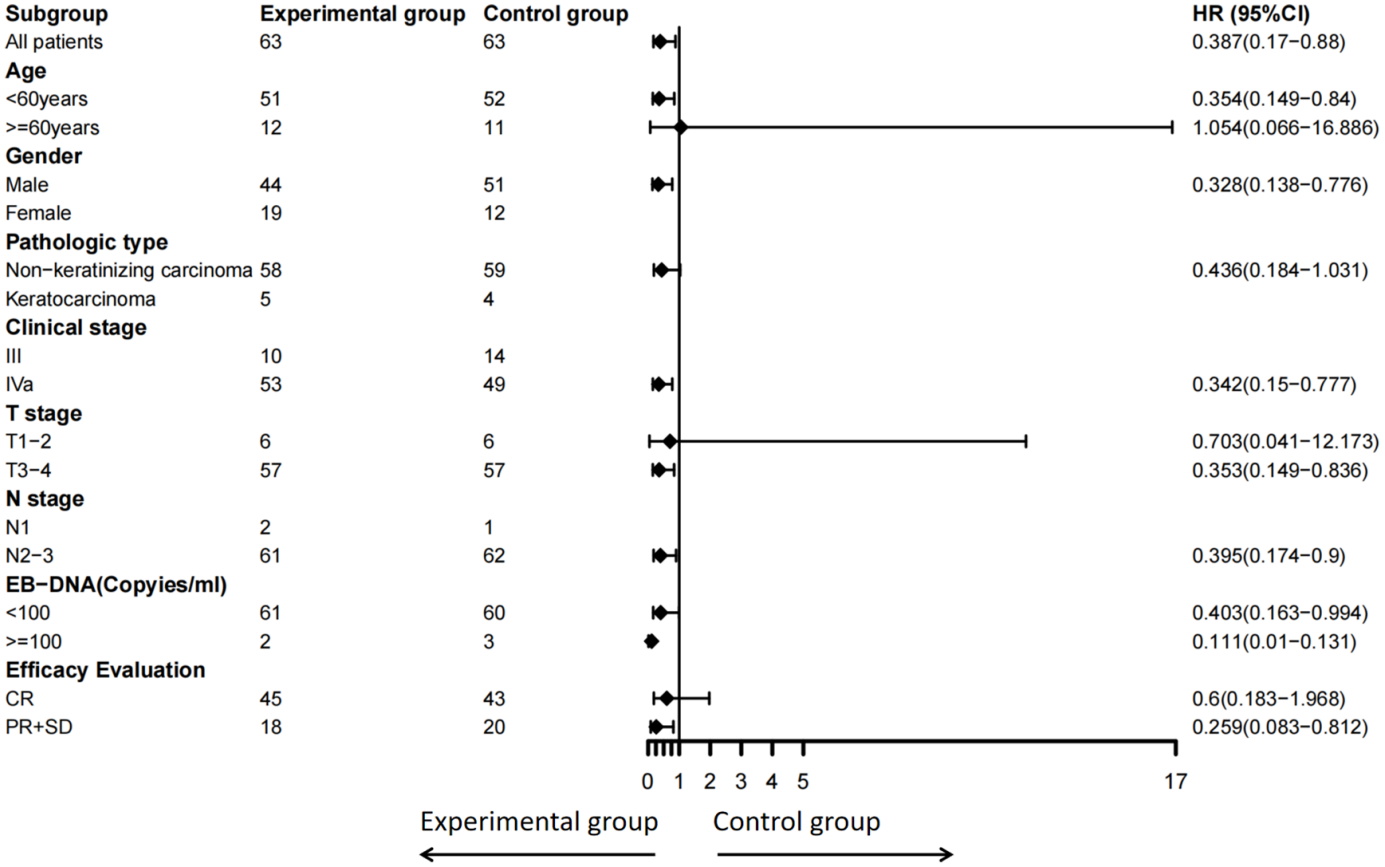
Subgroup analysis of DMFS by clinical variables including age, sex, pathologic type, clinical stage, T stage, N stage, EBV-DNA (copies/ml), and efficacy evaluation. DMFS, distant metastasis-free survival; EBV, Epstein-Barr virus. Due to the small sample size and few positive events in subgroups such as female, keratinizing carcinoma, clinical stage III, N1, the analysis results are exploratory and unstable.

Subgroup analysis showed that patients with high-risk locally advanced NPC who were under 60 years old (hazard ratio [HR]: 0.354, 95% confidence interval [CI]: 0.149–0.840, *P* = 0.018), male (HR: 0.328, 95% CI: 0.138–0.776, *P* = 0.011), had stage IVa disease(HR: 0.342, 95% CI: 0.150–0.777, *P* = 0.011), T3–4 tumors(HR: 0.353, 95% CI: 0.149–0.836, *P* = 0.017), or N2–3 disease (HR: 0.395, 95% CI: 0.174–0.900, *P* = 0.027), were EBV-DNA negative after chemoradiotherapy (HR: 0.403, 95% CI: 0.163–0.994, *P* = 0.048), or had either a partial response (PR) or stable disease (SD) after radical radiotherapy (HR: 0.259, 95% CI: 0.083–0.812, *P* = 0.020) had significantly improved DMFS following S-1 maintenance therapy. Patients with non-keratinizing carcinoma (HR: 0.436, 95% CI: 0.184–1.031, *P* = 0.058) or EBV-DNA positivity (HR: 0.111, 95% CI: 0.010–0.131, *P* = 0.063) also demonstrated a trend toward benefit in DMFS (**Figure 3**).

**Figure 4:**
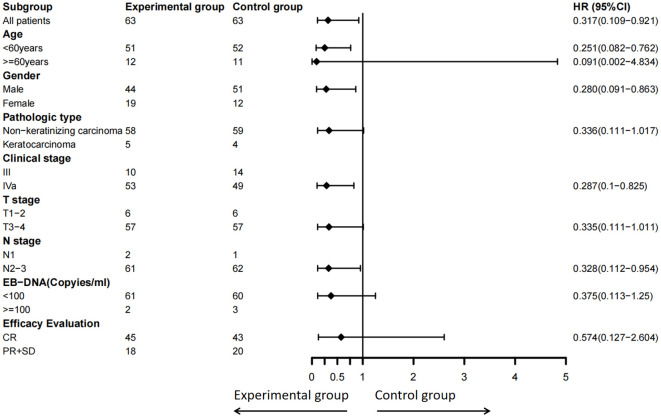
Subgroup analysis of OS by clinical variables including age, sex, pathologic type, clinical stage, T stage, N stage, EBV-DNA (copies/ml), and efficacy evaluation. OS, overall survival; EBV, Epstein-Barr virus. Due to the small sample size and few positive events in subgroups such as female, keratinizing carcinoma, clinical stage III, T1-2, N1, EB-DNA positive, PR+SD, the analysis results are exploratory and unstable.

Subgroup analysis also found that patients younger than 60 years old (HR: 0.251, 95% CI: 0.082–0.762, *P* = 0.014), male (HR: 0.28, 95% CI: 0.091–0.863, *P* = 0.026), those with stage IVa disease (HR: 0.287, 95% CI: 0.100–0.825, *P* = 0.021) or N2–3 disease (HR: 0.328, 95% CI: 0.112–0.954, *P* = 0.041), or patients who had PR or SD after radical radiotherapy (*P* = 0.020; HR not calculable due to no events in the experimental group) achieved significantly better OS after radical chemoradiotherapy followed by S-1 maintenance chemotherapy. Furthermore, patients with non-keratinizing carcinoma (HR: 0.336, 95% CI: 0.111–1.017, *P* = 0.053) or T3–4 disease (HR: 0.335, 95% CI: 0.111-1.011, *P* = 0.052) demonstrated a trend toward benefit in OS (**Figure 4**).

Multivariate Cox regression analysis demonstrated that EBV-DNA positivity after radical chemoradiotherapy was an independent risk factor for disease progression (HR: 6.690, *P* = 0.002), local recurrence (HR: 7.824, *P* = 0.005), distant metastasis (HR: 6.601, *P* = 0.002), and OS (HR: 4.279, *P* = 0.049). Patients with PR or SD after chemoradiotherapy were also at a higher risk of disease recurrence (HR: 6.690, *P* = 0.002). In contrast, S-1 maintenance chemotherapy significantly reduced the risk of distant metastasis (HR: 0.334, *P* = 0.027; **Table 2**).

**Table 2:** Cox multivariate regression analysis of prognostic factors for PFS, LRRFS, DMFS, and OS in the PSM cohort.

Variable	Hazard ratio (95% CI)	*P*
PFS		
Group (control group vs experimental group)	0.461 (0.193-1.101)	0.081
Gender (male vs female)	0.390 (0.088-1.731)	0.216
Age (<60 years vs ≥60 years)	0.511 (0.143-1.822)	0.301
Efficacy evaluation (CR vs PR+SD)	2.146 (0.923-4.989)	0.076
EBV-DNA (negative vs positive)	6.690 (2.002-22.350)	0.002
Pathology(Non-keratinizing type vs Keratinizing type)	1.195 (0.268-5.319)	0.815
Stage (Stage III vs Stage IVa)	273334.238 (0.000-9.108*10^176)	0.950
T stage (T1–2 vs T3-4)	0.401 (0.112-1.428)	0.158
N stage (N1 vs N2-3)	176796.935 (0.000-&)	0.980
LRRFS		
Group (control group vs experimental group)	0.733 (0.244-2.199)	0.580
Gender (male vs female)	0.281 (0.330-2.373)	0.244
Age (<60 years vs ≥60 years)	0.746 (0.174-3.191)	0.693
Efficacy evaluation (CR vs PR+SD)	2.773 (1.026-7.489)	0.044
EBV-DNA (negative vs positive)	7.824 (1.854-33.024)	0.005
Pathology(Non-keratinizing type vs Keratinizing type)	1.262 (0.228-6.989)	0.790
Stage (Stage III vs Stage IVa)	209375.483 (0.000-3.787*10^208)	0.959
T stage (T1–2 vs T3-4)	0.377 (0.100-1.422)	0.150
N stage (N1 vs N2-3)	140227.479 (0.000-&)	0.982
DMFS		
Group (control group vs experimental group)	0.334 (0.127-0.881)	0.027
Gender (male vs female)	0.480 (0.106-2.161)	0.339
Age (<60 years vs ≥60 years)	0.359 (0.079-1.642)	0.187
Efficacy evaluation (CR vs PR+SD)	2.229 (0.921-5.395)	0.076
EBV-DNA (negative vs positive)	6.601 (1.968-22.138)	0.002
Pathology(Non-keratinizing type vs Keratinizing type)	1.341 (0.295-6.085)	0.704
Stage (Stage III vs Stage IVa)	294327.631 (0.000-2.14*10^201)	0.956
T stage (T1–2 vs T3-4)	0.517 (0.113-2.362)	0.395
N stage (N1 vs N2-3)	179330.316 (0.000-&)	0.982
OS		
Group (control group vs experimental group)	0.316 (0.064-1.557)	0.316
Gender (male vs female)	0.522 (0.059-4.655)	0.560
Age (<60 years vs ≥60 years)	0.472 (0.077-2.892)	0.417
Efficacy evaluation (CR vs PR+SD)	2.455 (0.790-7.628)	0.121
EBV-DNA (negative vs positive)	4.279 (1.007-18.182)	0.049
Pathology(Non-keratinizing type vs Keratinizing type)	2.044 (0.350-11.932)	0.427
Stage (Stage III vs Stage IVa)	174579.937 (0.000-5.407*10^240)	0.965
T stage (T1–2 vs T3-4)	0.877 (0.106-7.228)	0.903
N stage (N1 vs N2-3)	124360.748 (0.000-&)	0.983

“&” indicates an infinite value (hazard ratio not calculable).Staging: Stage III vs Stage IVa and N staging: N1 vs N2–3 hazard ratio confidence interval is too large, which is due to the small sample size and number of positive events in subgroup Stage III and subgroup N1, leading to an imprecise estimate of the hazard ratio.

The asterisk (*) denotes multiplication, equivalent to the "×" symbol.The caret (^) denotes exponentiation, meaning "10 to the power of 240".

### Comparison of adverse reactions between the two groups

No grade 4 adverse reactions were observed in either group. There were two cases of grade 3 leukopenia in the experimental group and the overall incidence of leukopenia was significantly higher in this group than in the control group (74.6% vs. 55.6%, *P* = 0.025). The incidences of loss of appetite and skin pigmentation were also significantly higher in the experimental group compared to in the control group.

Regarding other adverse reactions, there were no significant differences between the two groups in other adverse reactions, such as hemoglobin and platelet levels, nausea, vomiting, oral mucositis, rash, diarrhea, fatigue, alanine and aspartate aminotransferase levels, and bilirubin and creatinine levels (**Table 3**).

**Table 3:** Comparison of adverse reactions between the two groups.

Adverse reactions	Experimental group (n=63)		Control group (n=63)		*P1*	*P2*
	Grade 1–2(%)	Grade 3–4 (%)	Grade 1–2(%)	Grade 3–4 (%)		
Leukopenia	45 (71.4)	2 (3.2)	35 (55.6)	0 (0.0)	0.064	0.476
Neutropenia	10 (15.9)	0 (0.0)	9 (14.3)	0 (0.0)	0.803	–
Hemoglobin decreased	29 (46.0)	0 (0.0)	24 (38.1)	0 (0.0)	0.367	–
Hypokalemia	5 (7.9)	0 (0.0)	4 (6.3)	0 (0.0)	1.000	–
Loss of appetite	13 (20.6)	0 (0.0)	4 (6.3)	0 (0.0)	0.006	–
Nausea	4 (6.3)	0 (0.0)	0 (0.0)	0 (0.0)	0.127	–
Vomiting	1 (1.6)	0 (0.0)	0 (0.0)	0 (0.0)	1.000	–
Skin pigmentation	12 (19.0)	0 (0.0)	1 (1.6)	0 (0.0)	0.001	–
Oral mucositis	4 (6.3)	0 (0.0)	1 (1.6)	0 (0.0)	0.361	–
Rash	1 (1.6)	0 (0.0)	0 (0.0)	0 (0.0)	1.000	–
Diarrhea	2 (3.2)	0 (0.0)	0 (0.0)	0 (0.0)	0.476	–
Fatigue	9 (14.3)	0 (0.0)	4 (6.3)	0 (0.0)	0.143	–
Increased ALT/AST	6 (9.5)	0 (0.0)	2 (3.2)	0 (0.0)	0.273	–
Increased bilirubin	4 (6.3)	0 (0.0)	2 (3.2)	0 (0.0)	0.676	–
Increased creatinine	1 (1.6)	0 (0.0)	1 (1.6)	0 (0.0)	1.000	

“-” indicates data not available for statistical analysis.*P1* is the *P* value of the Chi-square test for grade 1–2 adverse reactions in two groups, and *P2* is the *P* value of the Chi-square test for grade 3–4 adverse reactions in two groups.

## Discussion

This study showed that the 3-year DMFS and OS were significantly higher in the S-1 maintenance chemotherapy group than in the control group. This suggests that S-1 maintenance chemotherapy after radical chemoradiotherapy can reduce distant metastasis and improve OS in patients with high-risk locally advanced NPC.

A prospective phase II study found that IMRT combined with S-1 concomitant chemoradiotherapy achieved a high 3-year progression-free survival rate and overall survival rate in patients with locally advanced nasopharyngeal carcinoma (LANPC), with mild toxic reactions, providing solid clinical evidence for the application of S-1 in locally advanced nasopharyngeal carcinoma (16). Another multicenter retrospective study compared the efficacy and safety of S-1 with platinum-based concomitant chemoradiotherapy, showing that the S-1 group had comparable 5-year overall survival and progression-free survival rates to the platinum group, but with lower hematological and non-hematological toxicities (17). From a biological perspective, S-1 exerts a sustained antitumor effect by inhibiting thymidylate synthase and DNA synthesis (18), providing a rational basis for its use as maintenance therapy. Based on these findings, the application of S-1 not only offers an effective alternative for concomitant chemoradiotherapy but also provides a theoretical support for maintenance therapy.

S-1, as an oral fluoropyrimidine drug, can maintain a relatively stable drug concentration in the blood. The 5-fluorouracil (5-FU) produced after metabolism in the body can continuously inhibit the growth of micro-metastatic foci, which is particularly beneficial for clearing circulating tumor cells (CTCs) in the blood (19). Studies have shown that the presence of CTCs is closely related to the risk of distant metastasis. Changes in the number of CTCs before and after treatment can serve as efficacy predictive indicators (20). Although S-1 can effectively control systemic tumors, its penetration into the local area of the primary tumor may be limited, which may lead to the improvement of progression-free survival (PFS) and locoregional relapse-free survival (LRRFS) not reaching statistical significance. The study by Yu et al (21). further supports this view. The research found that the proportion of mesenchymal circulating tumor cells (mesenchymal CTCs, referring to the CTC subpopulation with a mesenchymal phenotype) is positively correlated with the degree of lymph node metastasis, and patients with fewer mesenchymal CTCs before neoadjuvant chemotherapy are more likely to achieve partial response (PR). In summary, we propose that S-1 may primarily improve distant metastasis-free survival (DMFS) and overall survival (OS) significantly by clearing CTCs and inhibiting the formation of micro-metastatic foci. However, its control effect on local lesions is relatively limited, which may be the main reason why PFS and LRRFS did not reach statistically significant differences.

Our subgroup analysis shows that patients with non-complete response (partial response/stable disease, PR/SD) benefit more significantly from S-1 maintenance therapy. We believe that these patients typically have a higher tumor burden and stronger tumor invasiveness, making them more sensitive to continuous systemic treatment. The study by Lu et al. (12) supports this view. The study shows that among patients with metastatic nasopharyngeal carcinoma, those with EBV-DNA positive status (associated with tumor burden and invasiveness) benefit more significantly from S-1 maintenance therapy.

Our results showed that 1 year of S-1 maintenance chemotherapy improved survival outcomes without increasing the incidence of severe toxicity. Consistent with our findings, Zong et al. (22) found improved 3-year OS and DMFS in high-risk patients receiving S-1 maintenance chemotherapy. However, their cohort included only patients with N3 lymph node stage and had treatment durations ranging from 3 to 24 cycles. Similarly, Lin et al. (23) demonstrated improved 4-year PFS, DMFS, and OS with S-1 maintenance chemotherapy in patients with N3 disease. High-risk locally advanced nasopharyngeal carcinoma maintenance therapy also includes the use of Capecitabine regimens or PD-1 inhibitors. Due to the lack of direct head-to-head studies, it is difficult to assess the efficacy differences. In terms of adverse reactions, the incidence of grade 3 adverse reactions in Capecitabine treatment is 17%, with common adverse events including hand foot syndrome, anemia, leukopenia, fatigue, and nausea (24). The incidence of grade 3 and above adverse reactions in Carrilizumab treatment is 11.2%, with common grade 3–4 adverse reactions including leukopenia and reactive capillary endothelial proliferation (25). In this study, the grade 3 adverse reaction for S-1 is leukopenia (3.2%), with other adverse reactions including loss of appetite and skin pigmentation. In terms of treatment costs, immunotherapy is the most expensive and is not reimbursed by medical insurance; while S-1 and Capecitabine have lower costs and are reimbursable by medical insurance. Therefore, in clinical treatment of patients, different drugs can be selected for maintenance therapy based on individual circumstances.

The optimal duration of maintenance chemotherapy for high-risk locally advanced NPC remains uncertain. Zheng et al. (26) found that 73% of metastatic events in locally advanced NPC occurred within 2 years post-radiotherapy. After this period, the risk of metastasis significantly declined. It remains uncertain whether a 1-year treatment duration is sufficient for these patients. In the study by Zong et al. (22), a number of maintenance chemotherapy cycles exceeding 5 was associated with improved survival. Meanwhile, Chen et al. (24) used a fixed 1-year duration of capecitabine maintenance chemotherapy. Furthermore, immunotherapy has also shown promise in advanced NPC (27).For example, Liang et al. (25) reported that 12 cycles of camrelizumab maintenance therapy improved 3-year event-free survival in locally advanced NPC.

In this study, multivariate regression analysis identified post-treatment EBV-DNA positivity as an independent prognostic factor for disease progression, recurrence, and poor prognosis, consistent with prior research findings (28–31). Based on this prognostic factor, high-risk patients with locally advanced NPC and persistently positive post-treatment EBV-DNA may require an intensive maintenance therapy regimen; for example,S1 combined with immunotherapy. The efficacy and safety of such intensive regimens warrant further investigation.

This study provides clinical evidence supporting the use of S-1 maintenance chemotherapy after radical chemoradiotherapy in high-risk, locally advanced NPC. Nevertheless, this study has some limitations. First, this is a retrospective study with potential selection bias. Second, our results are derived from a single-center cohort and may not be generalizable to patients from other institutions. In addition, due to the small sample size and positive events in certain subgroups (especially Stage III, N1), the risk ratio estimates for PFS, LRRFS, DMFS, and OS in the multivariate Cox regression analysis are not precise enough. Therefore, the interpretation of the analysis results should be cautious. Lastly, the follow-up duration is relatively short. Further studies that address these limitations should be conducted to validate our findings.

## Conclusion

S-1 maintenance chemotherapy after radical chemoradiotherapy significantly improves DMFS and OS, with mild and manageable adverse reactions. This regimen may be more suitable as adjuvant therapy for high-risk locally advanced nasopharyngeal carcinoma patients after definitive chemoradiotherapy, especially for patients with PR and SD after chemoradiotherapy.

## Data Availability

The raw data supporting the conclusions of this article will be made available by the authors, without undue reservation.
